# Clinical factors associated with severely reduced health status in patients with COPD and comorbid depression/anxiety: The Swedish PRAXIS study

**DOI:** 10.1038/s41533-026-00522-5

**Published:** 2026-05-16

**Authors:** Therese Öfverholm, Mikael Hasselgren, Karin Lisspers, Anna Nager, Gabriella Eliason, Maaike Giezeman, Christer Janson, Marta A. Kisiel, Scott Montgomery, Björn Ställberg, Josefin Sundh, Hanna Sandelowsky

**Affiliations:** 1 Department of Neurobiology, Care Sciences and Society, Division of Family Medicine and Primary Care, Karolinska Institutet, Stockholm, Sweden; 2 Academic Primary Care Centre, Region Stockholm, Sweden; 3 Centre for Clinical Research and Education Region Värmland, Karlstad, Sweden; 4 Department of Public Health and Caring Sciences, General Practice, Uppsala University, Uppsala, Sweden; 5 Department of Respiratory Medicine, Faculty of Medicine and Health, Örebro University, Örebro, Sweden; 6 School of Medical Sciences, Faculty of Medicine and Health, Örebro University, Örebro, Sweden; 7 Department of Medical Sciences, Respiratory, Allergy and Sleep Research, Uppsala University, Uppsala, Sweden; 8 Department of Medical Sciences, Occupational and Environmental Medicine, Uppsala University, Uppsala, Sweden; 9 Department of Occupational and Environmental Medicine, Akademiska Sjukhuset University Hospital, Uppsala, Sweden; 10 Clinical Epidemiology and Biostatistics, School of Medical Sciences, Faculty of Medicine and Health, Örebro University, Örebro, Sweden; 11 Division of Clinical Epidemiology, Department of Medicine, Karolinska Institutet, Stockholm, Sweden; 12 Department of Epidemiology and Public Health, University College London, London, UK

**Keywords:** Diseases, Health care, Medical research, Risk factors

## Abstract

Patients with COPD and comorbid depression/anxiety are at risk of reduced health status. This study aimed to assess health status in COPD patients with depression/anxiety, the occurrence of severely reduced health status in this group, and to investigate associations between severely reduced health status and patient-related clinical factors. This cross-sectional study included 2245 randomly selected COPD patients from primary care and hospital outpatient clinics in Sweden. The COPD Assessment Test (CAT) and the Clinical COPD Questionnaire (CCQ) were used to assess health status. Severely reduced health status was defined as CAT ≥ 20 or CCQ ≥ 2. The 524 patients (23.3%) who reported depression/anxiety had higher mean CAT scores (18.3 vs. 15.2, *p* < 0.001) and CCQ scores (2.37 vs. 1.83, *p* < 0.001) than patients without depression/anxiety. In this group, 48% had CAT ≥ 20 and 57% had CCQ ≥ 2. In patients with depression/anxiety, factors associated with CAT ≥ 20 were: onset of COPD symptoms before 60 years of age (OR = 2.62 [95% CI 1.37–5.04]), frequent symptoms of depression in the previous three months (2.59 [1.55–4.30]), one or more COPD exacerbations in the previous six months (2.37 [1.41–4.00]) and physical inactivity (1.89 [1.11–3.22]). Apart from physical inactivity, factors associated with CCQ ≥ 2 were the same as for CAT ≥ 20. Approximately half of the COPD patients with comorbid depression/anxiety reported severely reduced health status. Exacerbations, frequent depressive symptoms and physical inactivity were factors associated with severely reduced health status, and constitute important treatable and preventable traits of COPD.

## Introduction

Patients with chronic obstructive pulmonary disease (COPD) and comorbid depression and/or anxiety (hereafter referred to as “depression/anxiety”) have been identified as a high-risk group, with increased risk of exacerbations, hospitalization, and mortality^1–6^. These elevated risks may be related to a high symptom burden, although the relationship is incompletely understood^2,7^.

Health status can be defined in different ways. In COPD assessment, health status usually combines measures of symptoms, activity (functional status), and impact (psychosocial status)^8^. A simpler definition is based on a combination of symptom burden and quality of life^9^. Several tools have been developed to evaluate the impact of COPD symptoms on patients’ health and overall wellbeing, and two of the most widely used in clinical practice are the COPD Assessment Test (CAT)^10^ and the Clinical COPD Questionnaire (CCQ)^8^. Both instruments assess symptom burden^8,10^ and aspects of health-related quality of life (HRQoL)^11,12^, hence both can be used to measure health status. The CAT mainly focuses on COPD symptoms and functional level, whereas the CCQ is divided into three domains (symptoms, functional state, mental state), thus describing the impact of COPD in a more elaborated way^13^. Both tools have been found to correlate well with each other^13,14^. The Global Initiative for Chronic Obstructive Lung Disease (GOLD) recommends the use of the CAT as the preferred choice. However, the CCQ is described as an equal alternative^1,14^.

According to GOLD, patients with COPD should be assessed using the combination of airway obstruction, health status, and previous exacerbations, and categorized accordingly in the GOLD A-E system^1^. The choices of treatments and interventions should be based on the risk- and prognosis evaluations resulting from this combined assessment^1^.

Reduced health status is known to be associated with depression/anxiety in patients with COPD^15^. Previous studies have found that patients with depression/anxiety tend to score higher on CAT/CCQ, indicating worse health status, than those without depression/anxiety^12,15–18^. As reduced health status is associated with adverse outcomes, such as increased risk of exacerbations and mortality^19–21^, it is important to understand the factors that may contribute to its decline. However, it is still unclear how common severely reduced health status is in COPD patients with comorbid depression/anxiety. Moreover, it has not yet been explored whether certain patient characteristics are associated with reduced health status in this group.

The aims of this study were to assess health status in a cohort of COPD patients with depression/anxiety from Sweden, to assess the occurrence of severely reduced health status in this group, and to investigate associations between severely reduced health status and patient-related clinical factors in these patients.

## Materials and methods

### Design and data collection

The conducted study is part of the PRAXIS study, a large observational study of asthma and COPD in central Sweden (2005 – present)^22,23^. In this study, patients with COPD were randomly selected from 98 primary health care centers and 13 hospital clinics in 2014. Between 22 and 45 patients were selected from each site. Eligibility criteria were age over 18 years and having a doctor’s diagnosis of COPD (ICD–10 code J44) registered in the medical records within four years prior to recruitment. At the time of recruitment, postal invitations and informed consent forms were sent to 3877 patients, and reminders to non-responders were sent twice. The patients who had agreed to participate completed a patient questionnaire with information about their age, sex, exacerbation history, weight/height, educational level, physical activity, smoking habits, age of onset of COPD symptoms, pharmacological treatment of COPD, comorbidity including current or previous depression/anxiety, frequency of symptoms of depression/anxiety in the past three months, CAT, and CCQ.

### Definition of depression/anxiety

**Fig. 1:**
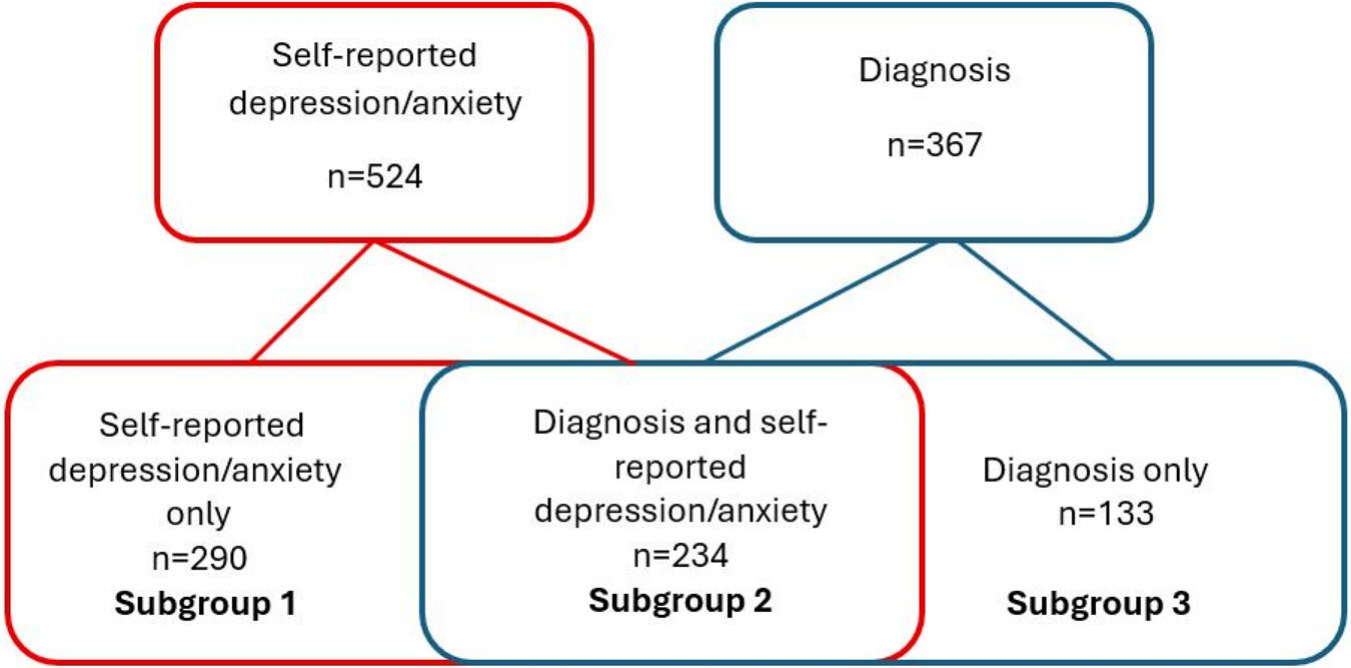
Flowchart of depression and anxiety identification and subgroup stratification. All patients in the cohort (n = 2245), divided into subgroups based on available data about depression and anxiety.

Self-reported information about comorbidity was provided through the patient questionnaire. Patients were determined to have self-reported depression/anxiety if they had answered “yes” to the following question: “Do you have/have had depression/anxiety?”. Medical record data on ICD-10 diagnoses of depression/anxiety (F32-F33, F41) registered between 2004 and 2014 were available to be extracted from medical records for 1267 patients (56%). Subgroup analyses were then performed based on available data on depression/anxiety. Three subgroups were identified: self-reported depression/anxiety only, self-reported and a diagnosis in medical records, and diagnosis in the medical records only (Fig. 1).

Our assumption was that the most reliable definition of patients with depression/anxiety would likely be subgroup 2 (self-reported depression/anxiety and a diagnosis in the medical records). We then tested our assumption by comparing subgroup 2 with subgroup 1 (self-reported depression/anxiety only; and subgroup 2 with subgroup 3 (diagnosis in the medical records only). When subgroups 1 and 2 were compared with each other, no significant differences in characteristics were found regarding age, gender distribution, GOLD stage, smoking status, education, BMI, level of dyspnoea measured by mMRC, exacerbation frequence, health status, and comorbidity. However, significant differences were found between subgroups 2 and 3, where those in subgroup 3 were significantly older, more often recruited from primary care, more often in GOLD stage 1 and stage 4, more physically active, less dyspnoic, more often had comorbid asthma, and reported significantly better health status. Furthermore, as patients in subgroup 3 did not report symptoms of depression/anxiety, yet they had a diagnosis of depression/anxiety registered in the medical records, we reasoned that the diagnoses were likely outdated.

As we concluded that subgroup 1 were very similar to subgroup 2, we included both these subgroups in the analysis as “patients with depression/anxiety” (n = 524).

### Variables

Lung function data from the most recently performed spirometry between 2004 and 2014 were extracted from the medical records. The forced expiratory volume in one second, percent of predicted (FEV_1_% pred.) was used to assess airflow limitation, and classified according to GOLD as stage 1 (mild) FEV_1_ ≥ 80% of predicted, stage 2 (moderate) 50% ≤ FEV_1_ < 80% of predicted, stage 3 (severe) 30% ≤ FEV_1_ < 50% of predicted, stage 4 (very severe) FEV_1_ < 30% of predicted^1^. Information on exacerbation history was collected via the patient questionnaire. The variable “exacerbation history” was dichotomized into no exacerbation or one or more exacerbations in the previous six months. A COPD exacerbation was defined as the need for treatment with an oral course of steroids and/or antibiotics due to worsening of respiratory symptoms, and/or to have made an emergency visit in primary care or at a hospital clinic due to worsening of respiratory symptoms. Body mass index (BMI) was calculated as kg/(m)^2^ and categorized as follows: underweight (BMI < 18.5), normal weight ( ≥ 18.5 BMI < 25), overweight ( ≥ 25 BMI < 30), and obesity (BMI ≥ 30). Educational level was dichotomized into lower or higher level of education, where higher level of education was defined as more than two years of education after nine years of compulsory school. Physical activity was dichotomized into being mostly inactive or active for at least four hours per week. Smoking status was dichotomized into never/former or occasional/daily smoker. The frequency of symptoms of depression in the previous three months was a variable that was dichotomized into always/most of the time or seldom/never. Age of onset of COPD symptoms was dichotomized into < 60 or ≥ 60 years. Information on medical treatment for COPD was collected through the patient questionnaire. The variable use of short-acting beta-agonist (SABA) was defined as having used SABA once or more in the previous week. The variable triple therapy was defined as having used regular treatment with a combination of inhaled corticosteroids (ICS), long-acting beta agonist (LABA), and long-acting muscarinic agonist (LAMA). Information about comorbidity was based on self-reported asthma, chronic bronchitis, allergies, heart disease, diabetes, stroke, hypertension, or cancer.

### Primary outcome measure: severely reduced health status

Severely reduced health status was assessed and defined by using the scores of CAT and CCQ. The CAT consists of eight questions that refer to symptoms of cough, sputum, chest tightness, breathlessness, limitations in activities, confidence in leaving the house, sleep, and energy. The scoring scale for each question ranges from 0 (best) to 5 (worst), with a total score ranging from 0–40. The CCQ consists of ten items divided into three domains: symptoms, functional state, and mental state. The scoring scale ranges from 0 (best) to 6 (worst) for each question, and the total score is the mean value of the ten items. The total score for each domain was calculated as the mean value of the domain’s questions. In the CCQ, patients are instructed to report their symptoms and perceived health status over the previous seven days, whereas in the CAT, patients are asked to describe their current state of symptoms. For both tools, the higher the score, the greater the negative impact on health status. In clinical practice, a CAT score > 10 indicates high symptom burden, and adjustment of treatment is considered necessary^1^. For even higher scores, CAT ≥ 17 is associated with increased risk of mortality^19^, and ≥ 20 with exacerbations^20^. Similarly, CCQ > 1 indicates unstable disease where treatment modifications are recommended, CCQ ≥ 2 indicates unstable disease for patients with severe COPD^8^, and CCQ ≥ 3 is associated with risk of mortality^21^. The minimum clinically important difference (MCID) for CAT is estimated to be two points^24^ and for CCQ to 0.4 points^25^.

In this study, severely reduced health status was defined as a CAT score ≥ 20 and a CCQ score ≥ 2.

### Statistical analysis

Standard parametric methods were used to assess summary statistics such as means, proportions, and measures of dispersion. Only patients who had completed all questions of the CAT or CCQ forms were included in the analysis. The level of statistical significance was defined as *p* value < 0.05. The CAT and CCQ scores were somewhat positively skewed, and a Shapiro-Wilk test confirmed that the data were not normally distributed (for CAT W = 0.98, *p* < 0.001, CCQ W = 0.96, *p* < 0.001). Parametric tests were still used to enable comparison with other studies, as it is standard procedure to compare means of CAT and CCQ. Therefore, in this study, the differences in the means of CAT and CCQ between patients with and without depression/anxiety were estimated by ANCOVA, with adjustment for age, sex, smoking, education, and lung function. A non-parametric Kruskal-Wallis test was also conducted to confirm the findings of the ANCOVA.

In the group of patients with depression/anxiety, differences in characteristics between patients with/without deteriorated health status were assessed by Chi^2^-test and T-test. Univariate logistic regression analyses were performed between deteriorated health status (CAT ≥ 20 and CCQ ≥ 2, respectively) and age, sex, exacerbation history, BMI, education, physical activity, smoking habits, and comorbidity. A multivariable logistic regression model was performed with the variables with statistically significant associations in the univariate analyses together with the variables age, sex, and lung function (FEV_1_% of predicted). No comorbidity showed a significant association with severely reduced health status in the final model and was therefore removed. Odds ratios (OR) and Confidence Intervals (CI) of 95% were provided. Statistical analyses were performed in SPSS software Version 28.0.1.1 (IBM Corp. Armonk, NY, USA) and STATA version 18.0 (StataCorp, College Station, TX, USA).

### Ethical approval

This study has been conducted in accordance with the principles of the Helsinki Declaration. Ethical approval was granted by the regional ethical board in Uppsala, Sweden, Dnr 2011/318, and the Regional Ethical Review Board of Stockholm, Dnr 2013/232-31/5.

## Results

**Fig. 2:**
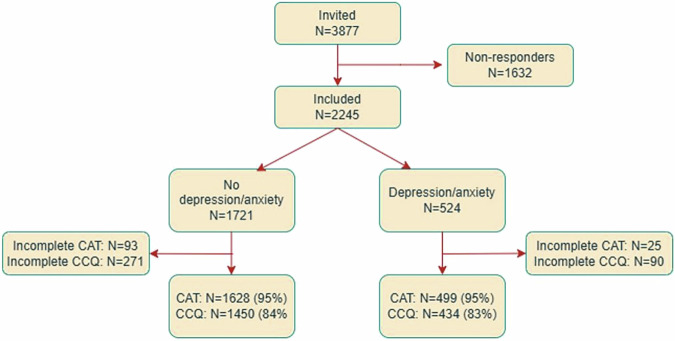
Flowchart of the study inclusion process. A flowchart of the study inclusion process and the response rate of CAT/CCQ for the included.

In total, 2245 patients replied with completed questionnaires and informed consent forms (response rate 58%). Of the responders, 524 (23.3%) reported current or previous depression/anxiety (Fig. 2). Of the non-responders, information on sex, age, level of care, and geographic area of recruitment was available in 1109 cases (67%). The non-responders were slightly younger, more often women (60% vs. 56%) and fewer were recruited from hospitals (21% vs. 26%) compared to the responders.

Of the 524 patients who reported depression/anxiety, 499 (95%) had completed the CAT and 434 (83%) the CCQ (Fig. 2). Patient characteristics of the total study population are available in Supplementary table 1.

**Fig. 3:**
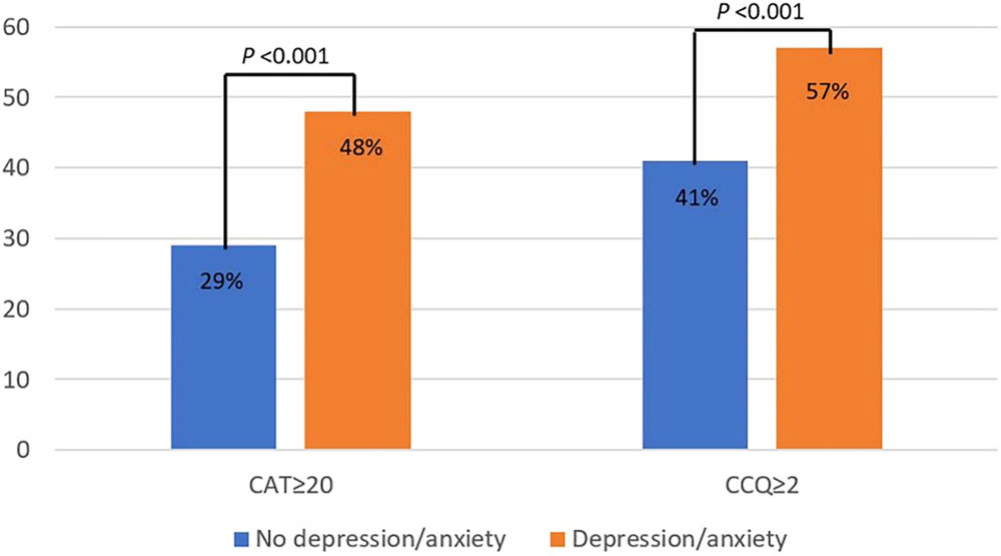
Severely reduced health status in the study population. Proportions of patients with severely reduced health status by the presence of depression/anxiety. All patients included. Blue – No depression/anxiety. Orenge – Depression/anxiety. CAT – COPD Assessment Test. CCQ – Clinical COPD Questionnaire.

Patients who reported depression/anxiety had higher mean CAT scores (18.3 vs. 15.2, *p* < 0.001) and CCQ scores (2.37 vs. 1.83, *p* < 0.001) than patients without depression/anxiety after adjustments for age, sex, smoking, education, and lung function. In patients with depression/anxiety, all domains of CCQ were elevated above the MCID compared to patients without depression/anxiety (Table 1). Severely reduced health status was more common in patients with depression/anxiety than those without depression/anxiety, measured by CAT ≥ 20 (48% vs. 29%, *p* < 0.001), and CCQ ≥ 2 (57% vs. 41%, *p* < 0.001) (Fig. 3).

**Table 1:** Mean scores of CAT^a^/CCQ^b^ with 95% CI^c^, adjusted for age, sex, smoking, education, and lung function.

Means (CI^c^)	No depression /anxiety Unadjusted	Current/ previous depression /anxiety Unadjusted		*p-value*	No depression /anxiety Adjusted	Current/ previous depression /anxiety Adjusted	*p-value*
CAT^a^	15.2 (14.8–15.6)		18.6 (17.9–19.3)	<0.001	15.2 (14.7–15.6)	18.3 (17.5–19.1)	<0.001
CCQ^b^, total	1.84 (1.78–1.90)		2.38 (2.27–2.50)	<0.001	1.83 (1.77–1.90)	2.37 (2.25–2.48)	<0.001
CCQ^b^, symptoms	2.07 (2.01–2.13)		2.46 (2.35–2.58)	<0.001	2.06 (2.00–2.13)	2.42 (2.30–2.54)	<0.001
CCQ^b^, functional state	1.78 (1.71–1.86)		2.24 (2.11–2.38)	<0.001	1.75 (1.68–1.83)	2.28 (2.14–2.41)	<0.001
CCQ^b,^ mental state	1.61 (1.53–1.69)		2.57 (2.43–2.72)	<0.001	1.60 (1.52–1.68)	2.54 (2.39–2.69)	<0.001

^a^CAT – COPD Assessment Test.

^b^CCQ – Clinical COPD Questionnaire.

^c^CI – Confidence interval.

The characteristics of patients exclusively with depression/anxiety, stratified by the level of their health status (i.e. CAT score < 20 or ≥ 20, and CCQ score < 2 or ≥ 2) are presented in Table 2. Patients with depression/anxiety and severely reduced health status (CAT ≥ 20/CCQ ≥ 2) had a more declined lung function, more often had experienced an exacerbation in the previous six months and were more physically inactive than those with lower CAT/CCQ scores. Moreover, it was more common in this group to have used SABA in the previous week, and to use triple therapy (ICS/LABA/LAMA). Information on comorbidity and BMI groups is available in Supplementary table 2.

**Table 2:** Characteristics of patients with depression/anxiety (N = 524) stratified by CAT^a^ < 20 or ≥ 20 and CCQ^b^ < 2 or ≥ 2.

Patient characteristics	CAT^a^ < 20 n = 262 (52.5)	CAT^a^ ≥ 20 n = 237 (47.5)	*p-*value	CCQ^b^ < 2 n = 187 (43.1)	CCQ^b^ ≥ 2 n = 247 (56.9)	*p-*value
Age, mean (SD^c^)	66.5 (7.3)	66.1 (8.0)	0.615	66.4 (6.9)	66.0 (8.1)	0.591
Sex, n (%)						
Women	187 (71.4)	165 (69.6)	0.668	139 (74.3)	173 (70.0)	0.325
FEV_1_^d^% predicted, mean (SD^c^)	62.0 (17.3)	53.7 (19.2)	<0.001	63.2 (16.2)	54.3 (19.6)	<0.001
GOLD^e^ stage, n (%)						
1	30 (13.0)	19 (9.3)	<0.001	25 (15.1)	19 (9.0)	<0.001
2	150 (65.2)	107 (52.5)		111 (66.9)	113 (53.3)	
3	42 (18.3)	55 (27)		28 (16.9)	56 (26.4)	
4	8 (3.5)	23 (11.3)		2 (1.2)	24 (11.3)	
Exacerbation previous six months, n (%)	62 (23.8)	130 (56.0)	<0.001	27 (14.6)	136 (55.7)	<0.001
BMI^f,^ mean (SD^c^)	26.8 (5.7)	26.9 (6.3)	0.848	27.0 (5.8)	26.9 (6.4)	0.842
Educational level, n (%)						
Lower	193 (75.4)	185 (80.1)	0.214	142 (76.8)	181 (75.1)	0.693
Physical activity, n (%)						
Mostly inactive	50 (25)	95 (51.4)	<0.001	36 (24.7)	87 (45.8)	<0.001
Smoking status, n (%)						
Current smoker	102 (39.7)	106 (46.1)	0.154	75 (40.5)	112 (45.7)	0.284
Symptoms of depression/anxiety the past three months						
Often/all the time	78 (38.6)	122 (61.3)	<0.001	43 (29.7)	126 (64.0)	<0.001
Age of onset of COPD symptoms, n (%)						
< 60 years	162 (67.2)	190 (80.5)	<0.001	112 (65.9)	191 (78.3)	0.005
Used SABA^g^ the past week, n (%)	102 (40.3)	153 (67.7)	<0.001	60 (33.1)	151 (64.3)	<0.001
Triple therapy^h^	76 (29.1)	141 (60.3)	<0.001	49 (26.5)	138 (56.6)	<0.001

^a^CAT – COPD Assessment Test.

^b^CCQ – Clinical COPD Questionnaire.

^c^SD – Standard Deviation.

^d^FEV_1_ – Forced expiratory volume of one second.

^e^GOLD – Global Initiative for Chronic Obstructive Lung Disease.

^f^BMI – Body mass index.

^g^SABA – Short-acting beta-agonist.

^h^Tripple therapy – ICS + LABA + LAMA.

**Fig. 4:**
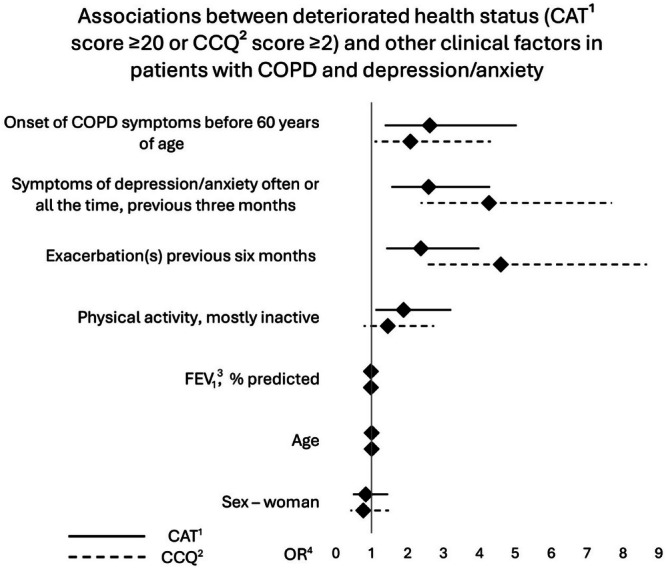
Clinical factors associated with severely reduced health status. Forest plot showing odds ratios (OR) and 95% confidence intervals (CI) for the associations between deteriorated health status (CAT¹ score ≥ 20 or CCQ² score ≥ 2) and other clinical factors in patients with COPD and comorbid depression/anxiety. All factors adjusted for each other in the analysis. ^1^CAT – COPD Assessment Test.^2^ CCQ – Clinical COPD Questionnaire.^3^ FEV_1_ – Forced expiratory volume of one second.^4^ OR – Odds Ratio.

Multiple logistic regression analysis, using CAT ≥ 20 as a response variable, revealed significant associations between CAT ≥ 20 and onset of COPD symptoms before 60 years of age (OR = 2.62 [95% CI 1.37–5.04]), frequent symptoms of depression in the previous three months (OR = 2.59 [95% CI 1.55–4.30]), COPD exacerbation(s) in the previous six months (OR = 2.37 [95% CI 1.41–4.00]) and physical inactivity (OR = 1.89 [95% CI 1.11–3.22]), after adjustments for all explanatory variables were made (Fig. 4). Factors associated with CCQ ≥ 2 were the same as for CAT ≥ 20, except for physical inactivity.

## Discussion

In this cross-sectional study of 2245 patients with COPD from primary care and hospital clinics in Sweden, patients with comorbid depression/anxiety had higher mean scores of CAT and CCQ compared to those without depression/anxiety. Severely reduced health status, defined as CAT ≥ 20 or CCQ ≥ 2, was more common in patients with depression/anxiety, and was associated with onset of COPD symptoms before 60 years of age, frequent symptoms of depression/anxiety in the previous three months, COPD exacerbation(s) in the previous six months and physical inactivity.

We found that the difference in mean scores between patients with and without depression/anxiety exceeded the MCID for both CAT^24^ and CCQ^25^, which is in accordance with previous studies^12,14,26^. Some have reported depression/anxiety to be one of the strongest factors associated with reduced health status^15^. Because of the known positive association between depression/anxiety and lower health status, one study investigated whether CAT could be used for screening of depression/anxiety in COPD patients, but this was not the case as the positive predictive value for a high CAT score was low^27^. However, in clinical practice, high CAT/CCQ scores may serve as an indicator that the patient should be assessed for psychological comorbidity according to current guidelines.

Interestingly, in our study, patients with depression/anxiety had higher CCQ scores in all domains (symptoms, functional state, mental state) compared to patients without depression/anxiety, with the largest difference in the mental state domain. This may be related to the generally high symptom burden in these patients, or high perception of symptoms of COPD^2,28^. As CAT and CCQ measure both symptom burden and aspects of health-related quality of life, other conditions that affect the patient’s experience of symptoms may affect the total scores^15^. For example, a previous study by the PRAXIS researchers reported heart disease, depression, and low BMI to be independently associated with reduced health status measured by both CAT and CCQ^14^. Hence, reduced health status may be caused by multiple factors, and an overall assessment of the patient is important.

In the present study, almost half (48%) of the patients with depression/anxiety reported significantly reduced health status measured by CAT, and more than half (57%) by CCQ. Still, we have not found other studies that have investigated the occurrence of severely reduced health status in patients with COPD and comorbid depression/anxiety. However, previous research show that reduced health status, measured by CAT and CCQ, is associated with increased risk of morbidity, exacerbations, and mortality^1–6^. In our study, approximately half of COPD patients with depression/anxiety may thus be at risk of severe negative clinical outcomes. This calls for attention and interventions from health care professionals working with these patients, since early identification and management is essential.

We found that patients with depression/anxiety and severely reduced health status used more SABA in the past week than those without depression/anxiety. This finding is in accordance with prior studies that show a strong association between dyspnea and anxiety^28,29^. SABA is prescribed to relieve symptoms of dyspnea, especially during an exacerbation^1^. In our study, exacerbations were, indeed, more common in patients with depression/anxiety. As anxiety is related to SABA overuse in asthma, this relationship may also apply to patients with COPD. One study found that overuse of SABA in COPD patients is associated with increased risk of exacerbations and mortality^30^. For the practicing clinician, it is important to be aware of frequent requests for SABA prescriptions and to evaluate health status and potential mental conditions in these patients.

In the present study, we found significantly reduced health status in COPD patients with depression/anxiety to be associated with the onset of COPD symptoms before 60 years of age, frequent symptoms of depression/anxiety, COPD exacerbation(s), and physical inactivity. To our knowledge, these associations have not been found previously. However, others have found that depression/anxiety is more common in younger patients with COPD^31,32^ and more common in patients with early, yet severe COPD than in older patients with milder disease^33^. Moreover, health status is known to deteriorate after an exacerbation^34,35^ and one study have found physical activity to decrease after an exacerbation^36^. Studies have also found physical activity to be reduced in patients with COPD and depression^37,38^. Hence there may be several factors contributing to the reduction of health status in these patients, and others have proposed theories that both physical and mental aspects may have synergizing effects in these patients, working in a bidirectional way to contribute to a spiral of declining health status^2,39^. A combined assessment of pulmonary and mental symptoms may thus be of importance in clinical practice, especially in patients with severely reduced health status.

Previous studies about the effects of treating depression/anxiety in patients with COPD are limited^40,41^, but a 2020 study from Norway found that the risk of mortality decreased when levels of anxiety improved^5^. Larger, prospective studies and randomized controlled trials on this subject are still too few for enabling definite conclusions^42^. As depression/anxiety clearly is associated with reduced quality of life and other negative clinical outcomes, it is an important treatable trait of COPD^43^ and should be taken into consideration when assessing a patient.

## Strengths and limitations

A strength of the study was its design with randomly selected patients from primary care and hospital clinics, as the study population, with a nearly equal distribution of the sexes, highly reflects the COPD population of Sweden^44,45^. Another strength of the study was that self-reported data from a real-world setting that provided the study with first-hand, up-to-date information about symptoms, medications, and perceived health status. As both CAT and CCQ primarily reflect disease-specific symptom burden and functional impact, and provide more detailed information about patients’ specific situations than data in a medical record, they serve well as indicators for overall health status in patients with COPD. As depression and anxiety are still underdiagnosed conditions in Sweden, first-hand information from patients was valuable because it enabled us to study patients that would have been missing in diagnose registers. Moreover, as depression/anxiety are dynamic conditions with high rates of remission (whether self-resolving or treated^46^), there is a risk that patients with outdated diagnoses are included if depression/anxiety is defined solely based on medical record diagnoses. Therefore, having first-hand information from the patients in combination with diagnoses from medical records was a strength of this study. However, there were some limitations in the study. Firstly, the use of self-reported data may have introduced bias in the reporting of depression/anxiety (recall bias). Secondly, the non-responders were slightly younger than the responders. Patients with more symptoms may thus have been more motivated to participate in the study (selection bias). On the other hand, in a previous questionnaire-based study from Sweden, a subgroup of non-responders, who were later contacted and interviewed, reported significantly more severe symptoms than the responders^47^.

Thirdly, the construction of the questionnaire may have led to limitations in the data. The CAT and the CCQ only collect information about symptoms of the previous week, hence the patient’s long term health status could not be assessed. Additionally, the questionnaire only provided information about exacerbations over the previous six months, thus preventing us from determining the GOLD A–E assessment of the participants. However, despite this limitation, we assessed that the available data enabled us to describe patients’ disease severity and burden sufficiently.

Finally, the data were collected more than ten years ago, which may have affected its current relevance. However, studies with more recent data have reported that the occurrence of depression/anxiety has remained largely unchanged in patients with COPD^48^, and improving health status still is central in COPD management^49^. As spirometry data were extracted from the most recent spirometry performed up to 10 years prior to the study, there is a risk of some patients having outdated spirometry data. Even though the data may be old, the lung function decline overtime is individual. We thus acknowledge this as a limitation, as some patients may have outdated spirometry data.

## Conclusion

In this study, nearly half of the patients with COPD and comorbid depression/anxiety had a severely reduced health status, which was associated with early onset of COPD symptoms, frequent symptoms of depression/anxiety, exacerbations, and physical inactivity. The results of this study call for increased awareness of and timely interventions against depression/anxiety in COPD patients, as it is an important treatable trait of COPD.

## Supplementary information

T Ofverholm Supplementary file 1

## Data Availability

The dataset generated and analyzed during the current study contains confidential information and is not publicly available due to ethical restrictions, but it is available from the corresponding author on reasonable request.

## References

[1] Global Initiative for Chronic Obstructive Lung Disease (GOLD). *Global strategy for the diagnosis, management, and prevention of chronic obstructive pulmonary disease*, http://www.goldcopd.org/ (2025).

[2] Pumar, M. I. et al. Anxiety and depression-Important psychological comorbidities of COPD. *J. Thorac. Dis.* **6**, 1615–1631 (2014). 25478202 10.3978/j.issn.2072-1439.2014.09.28 PMC4255157

[3] Maurer, J. et al. Anxiety and depression in COPD current understanding, unanswered questions, and research needs. *Chest* **134**, 43S–56S (2008). 18842932 10.1378/chest.08-0342 PMC2849676

[4] Papaioannou, A. I. et al. The impact of depressive symptoms on recovery and outcome of hospitalised COPD exacerbations. *Eur. Respir. J.* **41**, 815–823 (2013). 22878874 10.1183/09031936.00013112

[5] Vikjord, S. A. A., Brumpton, B. M., Mai, X. M., Vanfleteren, L. & Langhammer, A. The association of anxiety and depression with mortality in a COPD cohort. The HUNT study, Norway. *Respir. Med.* **171**, 106089 (2020). 32799059 10.1016/j.rmed.2020.106089

[6] Yohannes, A. M. et al. The association of depressive symptoms with rates of acute exacerbations in patients with COPD: results from a 3-year longitudinal follow-up of the ECLIPSE cohort. *J. Am. Med. Dir. Assoc.* **18**, 955–959 e956 (2017). 28733182 10.1016/j.jamda.2017.05.024

[7] Doyle, T. et al. Association of anxiety and depression with pulmonary-specific symptoms in chronic obstructive pulmonary disease. *Int. J. Psychiatry Med.* **45**, 189–202 (2013). 23977821 10.2190/PM.45.2.g PMC4005783

[8] van der Molen, T. et al. Development, validity and responsiveness of the Clinical COPD Questionnaire. *Health Qual. Life Outcomes* **1**, 13 (2003). 12773199 10.1186/1477-7525-1-13 PMC156640

[9] Jones, P. W. Health status measurement in chronic obstructive pulmonary disease. *Thorax* **56**, 880–887 (2001). 11641515 10.1136/thorax.56.11.880 PMC1745959

[10] Jones, P. W. et al. Development and first validation of the COPD Assessment Test. *Eur. Respir. J.* **34**, 648–654 (2009). 19720809 10.1183/09031936.00102509

[11] Gupta, N., Pinto, L. M., Morogan, A. & Bourbeau, J. The COPD assessment test: a systematic review. *Eur. Respir. J.* **44**, 873–884 (2014). 24993906 10.1183/09031936.00025214

[12] Sundh, J., Stallberg, B., Lisspers, K., Montgomery, S. M. & Janson, C. Co-Morbidity, Body Mass Index and Quality of Life in COPD Using the Clinical COPD Questionnaire. *Copd* **8**, 173–181 (2011). 21513436 10.3109/15412555.2011.560130

[13] Tsiligianni, I. G. et al. Assessing health status in COPD. a head-to-head comparison between the COPD assessment test (CAT) and the clinical COPD questionnaire (CCQ). *BMC Pulm. Med.* **12**, 20 (2012). 22607459 10.1186/1471-2466-12-20 PMC3431277

[14] Sundh, J. et al. Comparison of the COPD Assessment Test (CAT) and the Clinical COPD Questionnaire (CCQ) in a Clinical Population. *Copd* **13**, 57–65 (2016). 26367315 10.3109/15412555.2015.1043426

[15] Tsiligianni, I., Kocks, J., Tzanakis, N., Siafakas, N. & van der Molen, T. Factors that influence disease-specific quality of life or health status in patients with COPD: a review and meta-analysis of pearson correlations. *Prim. Care Respir. J.* **20**, 257–268 (2011). 21472192 10.4104/pcrj.2011.00029 PMC6549844

[16] Harryanto, H., Burrows, S. & Moodley, Y. A high COPD assessment test score may predict anxiety in COPD. *Int. J. Chron. Obstruct Pulmon Dis.* **13**, 955–957 (2018). 29606862 10.2147/COPD.S152950 PMC5868602

[17] Beech, A. & Singh, D. The COPD assessment test (CAT) and depression: a longitudinal analysis during the COVID-19 pandemic. *Int. J. Chron. Obstruct Pulmon Dis.* **18**, 1187–1195 (2023). 37332840 10.2147/COPD.S405050 PMC10276566

[18] Gudmundsson, G. et al. Depression, anxiety and health status after hospitalisation for COPD: a multicentre study in the Nordic countries. *Respir. Med.* **100**, 87–93 (2006). 15893921 10.1016/j.rmed.2005.04.003

[19] Casanova, C. et al. Differential effect of modified medical research council dyspnea, COPD assessment test, and clinical COPD questionnaire for symptoms evaluation within the new GOLD staging and mortality in COPD. *Chest* **148**, 159–168 (2015). 25612228 10.1378/chest.14-2449

[20] Lee, S. D. et al. The COPD assessment test (CAT) assists prediction of COPD exacerbations in high-risk patients. *Respir. Med.* **108**, 600–608 (2014). 24456695 10.1016/j.rmed.2013.12.014

[21] Sundh, J., Janson, C., Lisspers, K., Montgomery, S. & Stallberg, B. Clinical COPD questionnaire score (CCQ) and mortality. *Int. J. Chronic Obstr. Pulm. Dis.* **7**, 833–842 (2012). 10.2147/COPD.S38119 PMC3532021 23277739

[22] Ställberg, B. et al. Asthma control in primary care in Sweden: a comparison between 2001 and 2005. *Prim. Care Respir. J.* **18**, 279–286 (2009). 19455269 10.4104/pcrj.2009.00024 PMC6619358

[23] Arne, M. et al. How often is diagnosis of COPD confirmed with spirometry?. *Respir. Med.* **104**, 550–556 (2010). 19931443 10.1016/j.rmed.2009.10.023

[24] Kon, S. S. et al. Minimum clinically important difference for the COPD assessment test: a prospective analysis. *Lancet Respir. Med.* **2**, 195–203 (2014). 24621681 10.1016/S2213-2600(14)70001-3

[25] Kocks, J. W. et al. Health status measurement in COPD: the minimal clinically important difference of the clinical COPD questionnaire. *Respir. Res.* **7**, 62 (2006). 16603063 10.1186/1465-9921-7-62 PMC1508149

[26] Hilmarsen, C. W. et al. Impact of symptoms of anxiety and depression on COPD Assessment Test scores. *Eur. Respir. J.* **43**, 898–900 (2014). 24114965 10.1183/09031936.00163913

[27] Liu, M. S. et al. COPD Assessment Test as a Screening Tool for Anxiety and Depression in Stable COPD Patients: A Feasibility Study. *Copd.* **20**, 144–152 (2023). 37036434 10.1080/15412555.2023.2174843

[28] Regvat, J., Žmitek, A., Vegnuti, M., Košnik, M. & Šuškovič, S. Anxiety and depression during hospital treatment of exacerbation of chronic obstructive pulmonary disease. *J. Int. Med. Res.* **39**, 1028–1038 (2011). 21819737 10.1177/147323001103900338

[29] Neuman, A. et al. Dyspnea in relation to symptoms of anxiety and depression: a prospective population study. *Respir. Med.* **100**, 1843–1849 (2006). 16516455 10.1016/j.rmed.2006.01.016

[30] Janson, C., Wiklund, F., Telg, G., Stratelis, G. & Sandelowsky, H. High use of short-acting β(2)-agonists in COPD is associated with an increased risk of exacerbations and mortality. *ERJ Open. Res.* **9**, 00722–2022 (2023). 37342089 10.1183/23120541.00722-2022 PMC10277875

[31] Hanania, N. A. et al. Determinants of depression in the ECLIPSE chronic obstructive pulmonary disease cohort. *Am. J. Respir. Crit. Care Med.* **183**, 604–611 (2011). 20889909 10.1164/rccm.201003-0472OC

[32] Öfverholm, T. et al. High proportion of depression and anxiety in younger patients with COPD: a cross-sectional study in primary care in Sweden. *Scand. J. Prim. Health Care* **44**, 1–10 (2025). 40619171 10.1080/02813432.2025.2526667 PMC12918285

[33] Beijers, R., Franssen, F. M. E., Groenen, M. T. J., Spruit, M. A. & Schols, A. Physical and mental health profile of patients with the early-onset severe COPD phenotype: a cross-sectional analysis. *Clin. Nutr.* **41**, 653–660 (2022). 35131718 10.1016/j.clnu.2022.01.015

[34] Machado, A. et al. Impact of acute exacerbations of COPD on patients’ health status beyond pulmonary function: a scoping review. *Pulmonology* **29**, 518–534 (2023). 35715333 10.1016/j.pulmoe.2022.04.004

[35] Miravitlles, M. et al. Course of COPD assessment test (CAT) and clinical COPD questionnaire (CCQ) scores during recovery from exacerbations of chronic obstructive pulmonary disease. *Health Qual. Life Outcomes* **11**, 147 (2013). 23987232 10.1186/1477-7525-11-147 PMC3765881

[36] Esteban, C. et al. Change in physical activity related to admission for exacerbation in COPD patients. *Respir. Med.* **212**, 107236 (2023). 37023870 10.1016/j.rmed.2023.107236

[37] Duenas-Espin, I. et al. Depression symptoms reduce physical activity in COPD patients: a prospective multicenter study. *Int. J. Chronic Obstr. Pulm. Dis.* **11**, 1287–1294 (2016). 10.2147/COPD.S101459 PMC4910613 27354787

[38] Aldhahi, M. I. et al. Multifaceted associations between walking performance, physical fitness, extremity function, health status, and depression in individuals with COPD. *Ann. Med.* **56**, 2338248 (2024). 38590164 10.1080/07853890.2024.2338248 PMC11005873

[39] Yayan, J. & Rasche, K. Risk factors for depression in patients with chronic obstructive pulmonary disease. *Respir. Physiol. Neurobiol.* **315**, 104110 (2023). 37393968 10.1016/j.resp.2023.104110

[40] Fritzsche, A., Clamor, A. & von Leupoldt, A. Effects of medical and psychological treatment of depression in patients with COPD-a review. *Respir. Med.* **105**, 1422–1433 (2011). 21680167 10.1016/j.rmed.2011.05.014

[41] Kaplan, A. G. Do antidepressants worsen COPD outcomes in depressed patients with COPD?. *Pulm. Ther.* **10**, 411–426 (2024). 39516453 10.1007/s41030-024-00277-9 PMC11574234

[42] Pollok, J., van Agteren, J. E. & Carson-Chahhoud, K. V. Pharmacological interventions for the treatment of depression in chronic obstructive pulmonary disease. *Cochrane Database Syst. Rev.* **12**, Cd012346 (2018). 30566235 10.1002/14651858.CD012346.pub2 PMC6517114

[43] Agusti, A. et al. Treatable traits: toward precision medicine of chronic airway diseases. *Eur. Respir. J.* **47**, 410–419 (2016). 26828055 10.1183/13993003.01359-2015

[44] Backman, H. et al. Decreased COPD prevalence in Sweden after decades of decrease in smoking. *Respir. Res.* **21**, 283 (2020). 33115506 10.1186/s12931-020-01536-4 PMC7594463

[45] Carlsson, A. C. et al. High prevalence of diagnosis of diabetes, depression, anxiety, hypertension, asthma and COPD in the total population of stockholm, Sweden - a challenge for public health. *BMC Public. Health* **13**, 8 (2013). 23866784 10.1186/1471-2458-13-670 PMC3724714

[46] Malhi, G. S. & Mann, J. J. Depression. *Lancet* **392**, 2299–2312 (2018). 30396512 10.1016/S0140-6736(18)31948-2

[47] Rönmark, E., Lundqvist, A., Lundbäck, B. & Nyström, L. Non-responders to a postal questionnaire on respiratory symptoms and diseases. *Eur. J. Epidemiol.* **15**, 293–299 (1999). 10395061 10.1023/a:1007582518922

[48] Xie, H. et al. Global prevalence and risk factors of depression in patients with chronic obstructive pulmonary disease: a systematic review and meta-analysis from 2000–2022. *J. Psychosom. Res.* **175**, 111537 (2023). 37907038 10.1016/j.jpsychores.2023.111537

[49] Vogelmeier, C. F. et al. Goals of COPD treatment: focus on symptoms and exacerbations. *Respir. Med.* **166**, 105938 (2020). 32250871 10.1016/j.rmed.2020.105938

