# Supplementary material for: Clinical factors associated with severely reduced health status in patients with COPD and comorbid depression/anxiety: The Swedish PRAXIS study
Source: NPJ Prim Care Respir Med. 2026 May 16;36:29. doi: 10.1038/s41533-026-00522-5 (PMC13179948; doi:10.1038/s41533-026-00522-5)
Supplement: Supplementary file 1 — T Ofverholm Supplementary file 1 [file 41533_2026_522_MOESM1_ESM.pdf]

Supplementary file 1

Supplementary Table 1. Summary of characteristics of the whole study population and patients with and without depression/anxiety

Characteristics	Total study population<br>n = 2245	No depression /anxiety<br>n = 1721	Current/previous depression /anxiety<br>n = 524	*p-value*
**Age, mean (SD[1])**	68.6 (7.4)	69.3 (7.2)	66.5 (7.7)	<0.001
**Age categories, n (%)**				
<40	1 (0.043)	1 (100.0)	0	
40-49	31 (1.4)	16 (51.6)	15 (48.4)	
50-59	217 (9.7)	140 (64.5)	77 (35.5)	
60-69	892 (39.7)	651 (73.0)	241 (27.0)	<0.001
70-79	990 (44.1)	812 (82.0)	178 (18.0)	
80-89	105 (4.7)	92 (87.6)	13 (12.4)	
≥90	9 (0.4)	9 (100.0)	0	
**Gender, n (%)**				
Women	1269 (56.5)	899 (52.2)	370 (70.6)	<0.001
**FEV_1[2], percent of predicted, mean (SD)**	57.0 (18.6)<br>(Missing data=321)	56.8 (18.5)<br>(Missing data=252)	57.8 (18.6)<br>(Missing data=69)	0.296
**GOLD[3] stage, n (%)**				
1	197 (10.2)	147 (10.0)	50 (11.0)	0.055
2	1060 (55.1)	791 (53.8)	269 (59.1)	
3	537 (27.9)	433 (29.5)	104 (22.9)	
4	130 (6.8)	98 (6.7)	130 (6.8)	
**Exacerbation in the previous six months, n (%)**				
≥1	757 (34.2)	558 (32.9)	199 (38.5)	0.019
**BMI[4], mean (SD[1])**	26.5 (5.5)	26.3 (5.3)	26.9 (6.1)	0.035
**BMI[4] categories, n (%)**				
<18.5	110 (4.9)	79 (4.6)	31 (5.9)	0.023
18.5-24.9	822 (36.6)	647 (37.6)	175 (33.4)	
25-29.9	731 (32.6)	572 (33.2)	159 (30.3)	
≥30	582 (25.9)	423 (24.6)	159 (30.3)	
**Educational level, n (%)**				
Lower	500 (23.0)	382 (23.0)	118 (23.1)	0.995
**Physical activity, n (%)**				
Mostly inactive	536 (33.0)	381 (31.2)	155 (38.6)	0.006
**Smoking status, n (%)**				
Current smoker	702 (32.1)	482 (28.7)	220 (43.1)	<0.001
**Smoking, pack years, mean (SD[1])**	31.1 (18.9)	30.7 (18.9)	32.3 (18.8)	0.124
**Age of onset of COPD symptoms, n (%)**				
<60 years	1329 (62.6)	964 (59.4)	365 (73.0)	<0.001
**Used SABA[5] once or more last week, n (%)**	1011 (46.6)	745 (44.6)	266 (52.8)	0.001
**Triple therapy[6], n (%)**	955 (43.1)	728 (42.9)	227 (43.9)	0.685

[1] SD – Standard Deviation
[2] FEV_1 – Forced expiratory volume in one second
[3] GOLD – Global Initiative for Chronic Obstructive Lung Disease
[4] BMI – Body mass index
[5] SABA – Short acting beta agonist
[6] Tripple therapy – ICS + LABA + LAMA

Supplementary Table 2. Characteristics of patients with depression/anxiety (N=524) divided by CAT[1] <20 or ≥20 and CCQ[2] <2 or ≥2 – BMI[3] categories and comorbidity							
**Characteristics, n (%)**	**All with depression/ anxiety n = 524**	**CAT[1] <20 n = 262 (52.5)**	**CAT[1] ≥20 n = 237 (47.5)**	***p*-value**	**CCQ[2] <2 n = 187 (43.1)**	**CCQ[2] ≥2 n = 247 (56.9)**	***p*-value**
**BMI[3] categories**							
<18.5	31 (5.9)	12 (4.6)	17 (7.2)	0.366	7 (3.7)	17 (6.9)	0.471
18.5-24.9	175 (33.4)	91 (34.7)	77 (32.5)		66 (35.3)	81 (32.8)	
25-29.9	159 (30.3)	87 (33.2)	68 (28.7)		62 (33.2)	75 (30.4)	
≥30	159 (30.3)	72 (27.5)	75 (31.6)		52 (27.8)	74 (30.0)	
**Asthma**	172 (32.8)	83 (31.7)	83 (35.0)	0.429	50 (26.7)	93 (37.7)	0.017
**Chronic bronchitis**	43 (8.2)	12 (4.6)	29 (12.2)	0.002	9 (4.8)	26 (10.5)	0.030
**Allergies**	178 (41.8)	77 (37.2)	90 (45.2)	0.100	51 (34.0)	97 (49.2)	0.004
**Heart disease**	104 (19.8)	45 (17.2)	56 (23.6)	0.073	27 (14.4)	57 (23.1)	0.024
**Diabetes**	82 (15.6)	31 (11.8)	43 (18.1)	0.048	24 (12.8)	39 (15.8)	0.387
**Stroke**	47 (9.0)	21 (8.0)	23 (9.7)	0.506	10 (5.3)	25 (10.1)	0.070
**Hypertension**	226 (43.1)	112 (42.7)	43.9 (237)	0.799	77 (41.2)	112 (45.3)	0.386
**Cancer**	65 (12.4)	32 (12.2)	30 (12.7)	0.881	21 (11.2)	33 (13.4)	0.506

[1] CAT – COPD Assessment Test
[2] CCQ – Clinical COPD Questionnaire
[3] BMI – Body mass index
